# Post-outbreak dynamics and persistence of *Actinobacillus pleuropneumoniae* serotype 15 in finisher pigs in Iowa

**DOI:** 10.1186/s13567-025-01538-4

**Published:** 2025-05-27

**Authors:** Marcelo Nunes de Almeida, Pablo P. Pineyro, Derald Holtkamp, Isadora Machado, Ana P. S. Silva, Guilherme Cezar, Peter Thomas, Marcelo Gottschalk, Alyona A. Michael

**Affiliations:** 1 Department of Veterinary Diagnostic and Production Animal Medicine, College of Veterinary Medicine, Iowa State University, Ames, IA USA; 2 Iowa Select Farms, Iowa Falls, IA USA; 3 Groupe de recherche sur les maladies infectieuses en production animale (GREMIP), Faculté de médecine vétérinaire, Université de Montréal, Saint-Hyacinth, QC Canada; 4 Department of Pathobiology, College of Veterinary Medicine, Auburn University, Auburn, AL USA

**Keywords:** *Actinobacillus pleuropneumoniae*, tonsil scrapings, nasal swabs, environmental sampling, oral fluids, persistence, swine

## Abstract

In 2021, *Actinobacillus pleuropneumoniae* serotype 15 (App15) mediated a high mortality respiratory outbreak in finisher hogs, affecting multiple companies within a 30-km radius of Iowa, USA. The atypical regional spread raised concerns for the strain’s unusual environmental persistence and survivability. This prospective longitudinal study aimed to determine the duration of App15 persistence in convalescent pigs at a naturally infected 1200 head site. Sixty-seven pigs were sampled individually for 6 weeks using nasal swabs (NS), tonsil scrapings (TS), and serum samples (SS); pen-based oral fluids (OF) were also collected. NS, TS, and OF were tested for App by rtPCR, while serum was screened with the Swinecheck mix-App ELISA. All 67 pigs tested App PCR positive in TS ≥ 1 during the sampling period, with progressive increases in detection from 53.3% to 95.8% between the first and last sampling week, respectively. Only 23 pigs (34.3%) were PCR positive by NS ≥ 1 during the sampling period, with decaying detection rates from a peak of 51.2% positivity in the first sampling week. Fifty-three of 73 pens (72.6%) tested App PCR positive in OF ≥ 1 during the sampling period. Seropositivity decreased from 93% on week 4– to 33% on week 8. TS had the highest PCR detection rate at all time points evaluated, representing the best antemortem sample type for App PCR detection in this study. The results reported here generated new important knowledge related to App15 ecology and epidemiology, prospectively informing disease diagnosis, surveillance, and biosecurity practices.

## Introduction

*Actinobacillus pleuropneumoniae* (App) is a Gram-negative facultatively anaerobic coccobacillary bacterium and the etiologic agent of porcine pleuropneumonia [1]. App is subclassified into 19 known serotypes [2], which vary in pathogenicity depending on the expression of virulence factors, including a combination of Apx toxins, capsule polysaccharides, and endotoxins, among others [3, 4]. Introducing a virulent strain into susceptible populations results in the rapid onset of high fever (41 °C), coughing, and respiratory distress. Lung infection induces profound tissue necrosis and hemorrhage driven by vascular insult and exacerbated by the host’s exuberant inflammatory response; resultant fatalities often present with characteristic bloody and foamy nasal discharge. Epidemiologic outcomes of field infections can vary widely, depending on the involved strain [5], degree of host immunity [6], and extant coinfections [7]. Therefore, morbidity and mortality can range widely, from 10–100% and 1–10%, respectively [8, 9]. Economic losses continue to accrue post-outbreak in the form of reduced average daily gain [10] and elevated rates of carcass trimming and condemnation in convalescent pigs [11]. Transmission has traditionally been associated with direct oral or nasal contact between carriers [12] and, to a lesser degree, aerosolized respiratory droplets over short distances [13, 14]. Canonically, indirect transmission is thought to be negligible. However, both experimental and field investigations have implicated wind- and fomite-borne transmission between sites over short distances, from 400 m to 3 km [15–17]. App is considered an obligate parasite with poor environmental persistence; however, survival can be extended in organic material, wet environments, and cool ambient temperatures [18]. Furthermore, the formation of biofilms and persistence in waterers has been documented [19].

Virulent strains of App associated with high mortality outbreaks are uncommon in the United States [5], and vaccination is not routinely practiced in commercial operations in this country. Sporadic low-mortality outbreaks can occur in the later finisher phase within endemically infected systems as maternal immunity to persistent strains wanes and pigs are exposed to immunosuppressive stressors and comorbidities [20].

Diagnostic modalities commonly employed to directly detect App include bacteriology and PCR, with antibody-based tests, used to detect exposure to the bacterium [4]. Antibodies can be detected 7–14 days post exposure to App with increasing titers in the next few weeks and persistence for months [21]. However, experimental studies have demonstrated that not all pigs challenged with App may develop an antibody response, and that PCR detection from tonsillar scrapings is considered the most sensitive method for identifying positive animals using antemortem samples [22–24].

In late November 2021, several finisher sites from 9 unrelated systems in central Iowa (IA) began reporting severe App outbreaks, with mortality rising up to 51% over a matter of days. Notably, affected sites were geographically clustered within a 20-mile radius in central IA, and isolates were identified as App serotype 15 (App15) [25]. At the Iowa State University Veterinary Diagnostic Laboratory (ISU-VDL), the most commonly detected serotypes historically are App7 and App8. App serotype 15 is associated with high virulence in Australia [26], but is infrequently isolated and not commonly reported to cause high mortality outbreaks in the United States [27].

This outbreak challenged two paradigms of App dynamics in the United States: 1) low prevalence of virulent strains, and 2) minimal risk for App lateral transmission in clinical outbreaks cases. In the absence of a known origin or mechanism for the unusual persistence in the area, App15 remains a threat to swine producers. Therefore, this study aimed to characterize post-outbreak shedding and subsequent persistence of App15 under field conditions.

## Materials and methods

### Study design

This prospective longitudinal study was conducted at one IA finisher site, six weeks post placement (110 days of age), following a confirmed natural App15 outbreak on 30/12/2021 [25]. Sick pigs were mass treated with ceftiofur sodium as prescribed by the manufacturer. Individually identified pigs were repeatedly sampled weekly to evaluate App15 persistence in the nasal cavity and tonsils, and monitor the development of humoral immunity starting 3 and 4 weeks post-outbreak. The same pigs and pens were sampled weekly for the duration of the study. These data were correlated to population shedding dynamics and environmental persistence in the form of pen-based oral fluid (OF) and environmental samples.

### Farm

The study was conducted at a finisher site located in IA following a natural App15 outbreak. This site had three barns, each holding ~1200 pigs in 48 adjustable pens of variable sizes. Pens were arranged in a way that barns 1, 2, and 3 contained 25, 27, and 25 pens each, respectively. One and three pens were empty in barns 2 and 3, respectively, resulting in barns 1, 2, and 3 having 25, 26, and 22 pens with pigs, respectively.

### Samples

One pig per each pen containing ≥ 5 individuals (*n* = 67 pigs total) was uniquely identified via numbered ear tags to facilitate the weekly collection of nasal swabs (NS), tonsil scrapings (TS), and serum samples. OFs were collected as previously described [28] from all pens regardless the number of pigs (*n* = 77) in barns 1, 2, and 3. In brief, one 100% cotton rope was secured in each pen, with the end of the rope hanging at the height of the pigs’ shoulders. Pigs were allowed to chew on the ropes for 20–30 min after which absorbed OFs were harvested by placing the wet end of the rope in a plastic bag and extruding fluid by running the rope through clenched fingers. The fluid that was pooled in the bag was then poured into a 50 mL conical centrifuge tube (Thermo Fischer Scientific™, Pittsburgh, PA, USA).

Primary environmental persistence sampling took place at six feeders per barn, selected in accordance with a spatially distributed systematic sampling procedure [38]. Surface samples included feeder nipple, feeder plate, and floor in front of feeders. These were collected using a sterile 3 × 3″ gauze (AmerisourceBergen, Chesterbrook, PA, USA) pre-wetted with 5 mL of 20 mM phosphate buffer (PBS) solution [29]. Water samples from nipples were collected at each feeder. Additional environmental samples from each barn included water from a main line faucet in the entrance of each barn, the floor at the entrance of the barn, and the outside door handle giving access to the barn. External environmental samples included the rendering area and office door handle.

### Sample handling

Samples were maintained at 4° to 8 °C and transported to the diagnostic laboratory for processing within 24 h. In the laboratory, whole blood was centrifuged for 10 min at 1600 × *g*, and then serum samples were submitted for testing.

### Diagnostic testing

Serum samples were tested for App antibodies by enzyme-linked immunosorbent assay (ELISA) using the Swinecheck mix-APP 1–2–9–11, 3–6–8–15, 4–5–7, and 10–12 as previously described [30]. Results were expressed as corrected sample-to-positive (S/P) ratio. Samples were considered to be positive if the S/P ratio was equal to or greater than 0,5; samples with results below the positive cutoff but equal or greater than 0,3 were considered to be suspect; samples with S/P ratio less than 0,3 were considered negative. TS, NS, OFs, and environmental samples were individually tested by rtPCR as described elsewhere [31]. Bacteriology optimized for App isolation was individually performed on environmental samples that tested positive on PCR.

### Data analysis

Descriptive statistics were performed for all sample types and tests performed. Crude agreement at the week level for different sample types at the animal and pen-level were reported.

## Results

**Figure 1:**
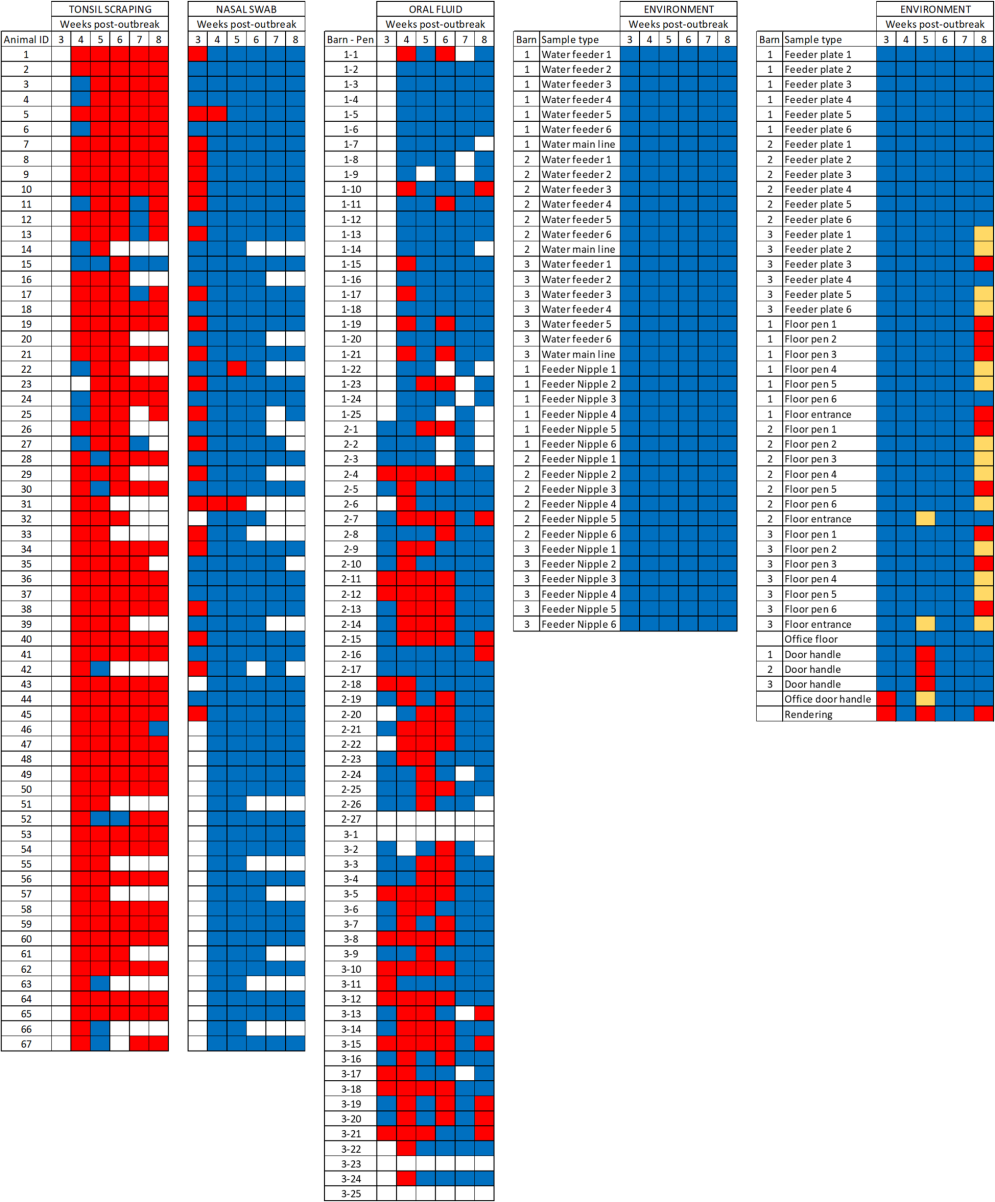
**Spatiotemporal patterns of Actinobacillus pleuropneumoniae detection in tonsil scrapings, NS, and OFs by weekly PCR testing starting 3 weeks post-outbreak**. Red represents a positive PCR result; blue represents a negative PCR result; yellow represents a suspect PCR result. White represents an empty pen, not sampled (pen or animal), or not available for sampling (pig not in the barn).

**Figure 2:**
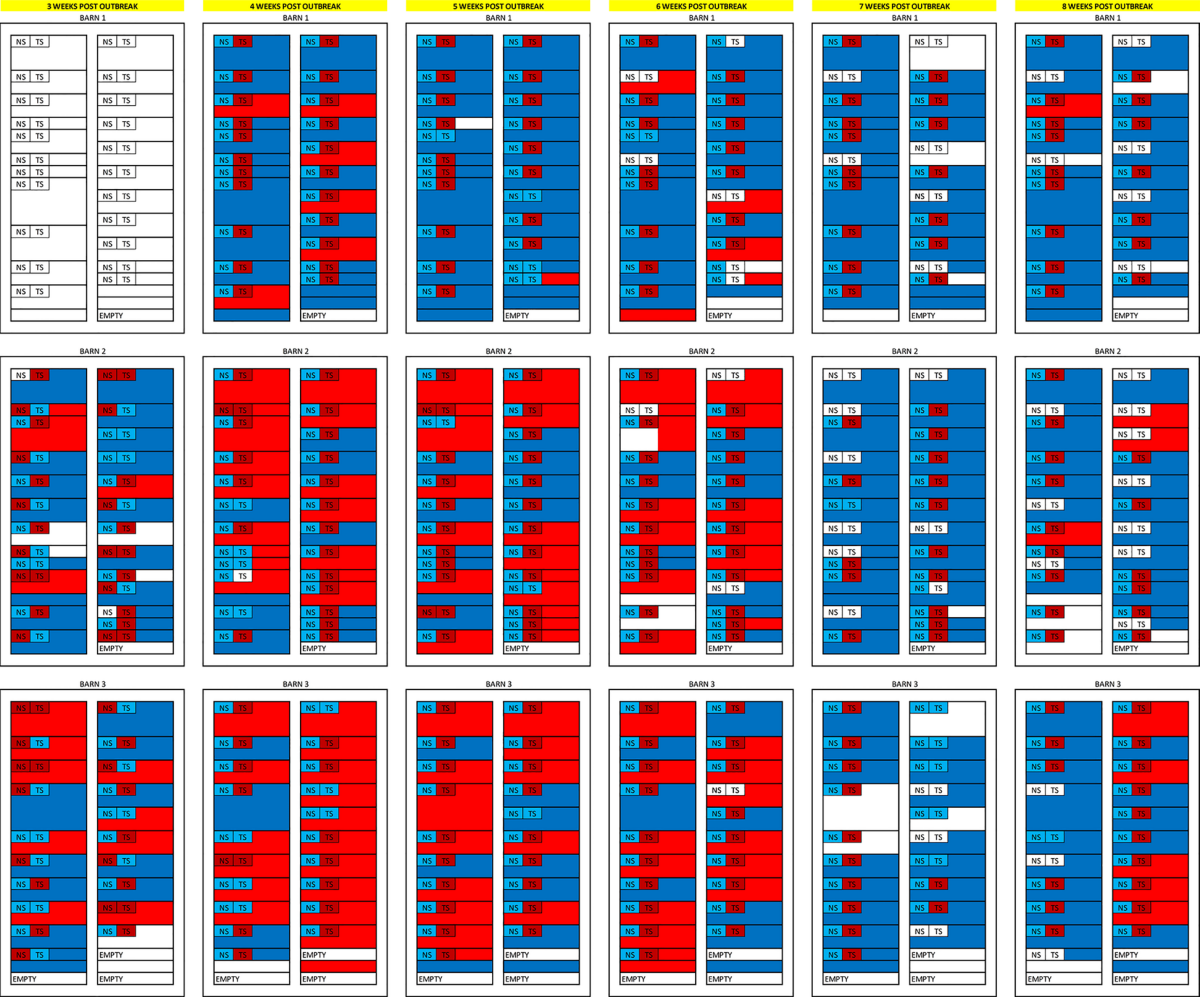
**Actinobacillus pleuropneumoniae DNA detection (red represents a positive PCR, blue represents a negative PCR) by barn, pen, and pig assessed by oral fluids (pen-level), nasal swabs, and tonsil scrapings (individual level).** NS = nasal swab, TS = tonsil scrapings. Large rectangles represent pens within barns.

App PCR detection over time in a three-barn site is summarized in Table 1. Detection in NS was highest at the first sampling point, with 51.2% of samples testing positive, with a sharp decline in detection on the second and third time points, and no detection in the remaining three sampling points. Comparatively, tonsil samples were positive in a similar proportion at the first sampling point with an increasing positivity rate until peak detection in sampling point 4 and a slight decrease at the last week of sampling with 95.8% detection rate. OF samples had the highest detection rate at the second sampling point with 54.2%, but notably, only 9 OF samples (14.1%) were found positive at the last sampling point. All results are depicted in Figures 1 and 2.

**Table 1:** **Summary of App detection by PCR in nasal swabs, tonsil scrapings, and oral fluids over time per barn and overall**

	Sampling point	Nasal swab # positive/# tested (percentage)	Tonsil scrapings # positive/# tested (percentage)	Oral fluid # positive/# tested (percentage)
Barn 1	1	–/–	–/–	–/–
	2	0/22 (0.0)	22/22 (100.0)	6/25 (24.0)
	3	0/22 (0.0)	18/22 (81.8)	1/24 (4.2)
	4	0/18 (0.0)	15/16 (93.8)	5/23 (21.7)
	5	0/16 (0.0)	16/16 (100.0)	0/20 (0.0)
	6	0/16 (0.0)	16/16 (100.0)	1/21 (4.8)
Barn 2	1	12/23 (52.2)	14/25 (56.0)	4/23 (17.4)
	2	1/25 (4.0)	20/24 (83.3)	16/26 (61.5)
	3	2/25 (8.0)	22/25 (88.0)	16/26 (61.5)
	4	0/22 (0.0)	22/22 (100.0)	14/24 (58.3)
	5	0/17 (0.0)	15/16 (93.8)	0/25 (0.0)
	6	0/15 (0.0)	15/15 (100.0)	3/22 (13.6)
Barn 3	1	10/20 (50.0)	10/20 (50.0)	9/20 (45.0)
	2	1/20 (5.0)	14/20 (70.0)	17/21 (81.0)
	3	0/20 (0.0)	19/20 (95.0)	13/22 (59.1)
	4	0/19 (0.0)	19/19 (100.0)	14/22 (63.6)
	5	0/17 (0.0)	12/17 (70.6)	0/20 (0.0)
	6	0/17 (0.0)	16/17 (94.1)	5/21 (23.8)
Overall	1	22/43 (51.2)	24/45 (53.3)	13/43 (30.2)
	2	2/67 (3.0)	56/66 (84.8)	39/72 (54.2)
	3	2/67 (3.0)	59/67 (88.1)	30/72 (41.7)
	4	0/59 (0.0)	56/57 (98.2)	33/69 (47.8)
	5	0/50 (0.0)	43/49 (87.8)	0/65 (0.0)
	6	0/48 (0.0)	46/48 (95.8)	9/64 (14.1)

All 67 pigs tested App PCR positive in tonsil scrapings at least once during the sampling period. Twenty-five pigs (37.3%) were positive in all time points (11 pigs in 6 time points, and 14 pigs in 5 time points) using tonsil scrapings, while using NS none of the pigs sampled were positive at all sampling points during the sampling period. Only 23 pigs (34.3%) were positive in NS at least once during the sampling period. One pig was positive in three sampling points, another pig was positive in 2 sampling points, and 21 pigs were only positive in one sampling point using NS. Forty-six of 48 pigs (95.8%) were App DNA positive in tonsil scraping at the last time point, 8 weeks after the reported outbreak. All forty-eight pigs tested negative for App DNA in NS at the last time point. Fifty-three of 73 pens (72.6%) tested App DNA positive in OFs at least once during the sampling period. Fifteen pens (20.5%) only tested positive once. Thirty-eight pens tested positive > 2 time points, of which 14 showed an intermittent detection pattern (1 positive results followed by a negative result followed by a positive result; or, 2 or 3 consecutive positive results followed by a negative result followed by a positive result). Of the pens showing consecutive detection of App DNA in OFs 11, 5, and 8 pens had 2, 3, and 4 consecutive positive results, respectively.

Regarding environmental sampling, at the first sampling point only the rendering box (dead animal collection point) and the office door handle tested positive for App by PCR. The rendering box was positive again in the third time point, and the door handles of all three barns on site also tested positive; the office door handle, and the floor by the entrance door of barns 2 and 3 tested as suspect by PCR at the third time point. At the last sampling time point 11 samples tested positive for App by PCR (rendering box, one feed plate at barn 3; 3, 2, and 3 floor samples collected in front of feeders in barns 1, 2, and 3, respectively, and the floor by the entrance door of barn 1). Additionally, 13 samples tested as suspect on PCR (3 feed plates in barn 3; 2, 4, and 3 floor samples collected in front of feeders in barns 1, 2, and 3, respectively, and the floor by the entrance door of barn 3). Throughout sampling points, a partial necropsy was performed in dead pigs in rendering box. The majority of necropsied pigs had residual lung damage macroscopically consistent with App infection. All environmental samples tested negative for App by PCR on the second, forth, and fifth time points (Figure 1). Positive samples were submitted for culture, but failed to yield viable isolates.

**Figure 3:**
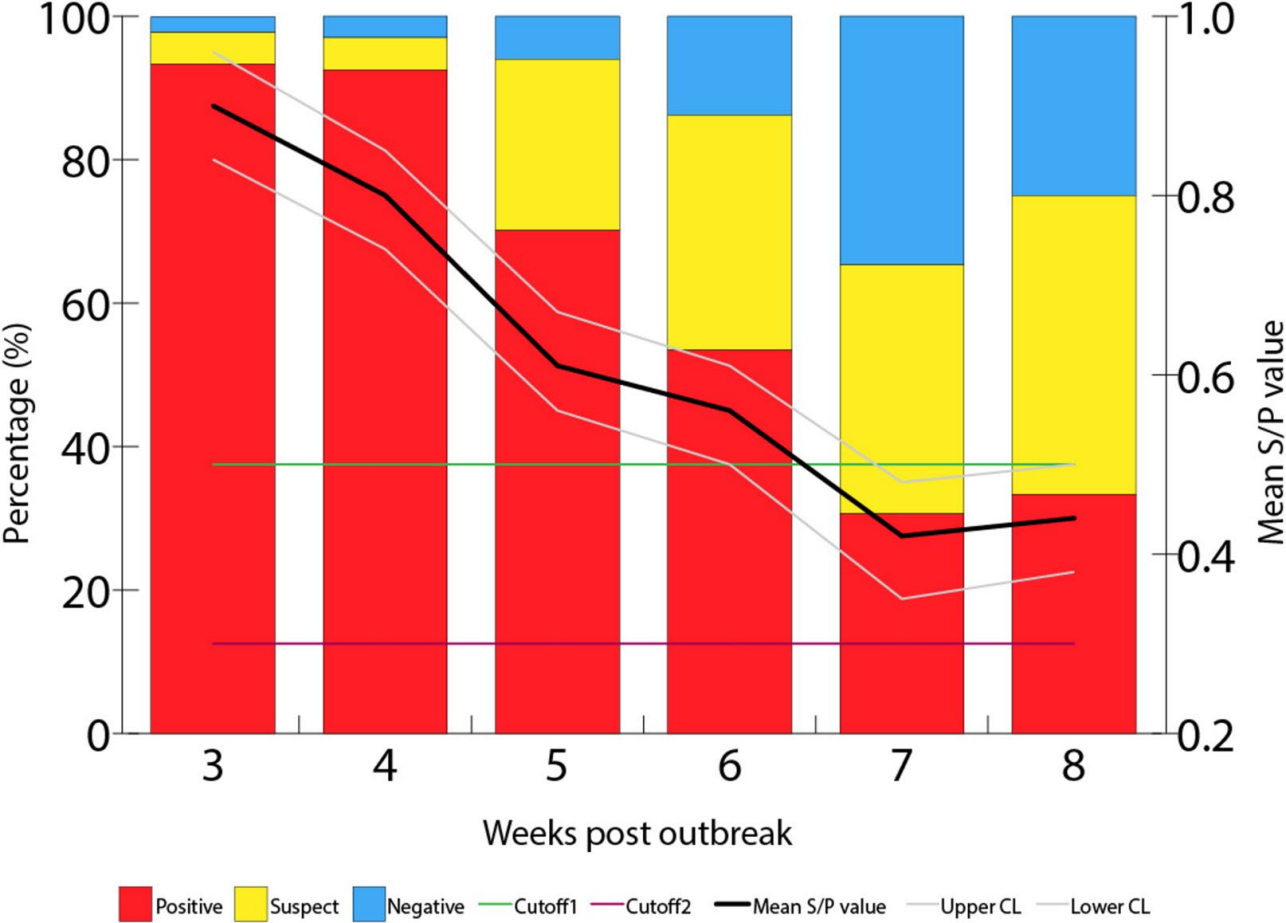
**Percentage of pigs ELISA positive for App 3–6–8–15 and average S/P values for App ELISA serogroup 3–6–8–15 and 95% confidence intervals.** Cutoff1 and cutoff 2 were 0.3 and 0.5, respectively, representing the threshold below which the samples were considered negative and above which the samples were considered positive. Values between cutoff1 and cutoff 2 were considered suspect.

Both the average App ELISA S/P values and proportion of positive pigs decreased over time during the sampling period. The percentage of App ELISA positive pigs for serogroup 3–6–8–15 was 93.3% (42 of 45), 92.5% (62 of 67), 70.1% (47 of 67), 52.5% (31 of 59), 30.6% (15 of 49), and 33.3% (16 of 48) at 3, 4, 5, 6, 7, and 8 weeks post-outbreak, respectively (Figure 3).

## Discussion

*Actinobacillus pleuropneumoniae* is an important bacterial component of the porcine respiratory disease complex that can exhibit dramatic epizootic clinical presentations with high death loss. This agent was first isolated in the United States in the late 1950 s and beginning of 1960 s [32, 33]. An increased incidence of App was recognized in in the late 1970 s with the ISU-VDL reporting cases of the disease confirmed by culture increasing from 2 cases in 1976 to 9, 7, 33, and 69 for 1977, 1978, 1979, and 1980, respectively. Serological screening of Iowa sow herds in 1982 demonstrated 68.8% seropositivity for App serotypes 1–5 [34]. The annual rate of clinical App cases appears to have remained steady over the last four decades at the ISU-VDL, averaging 79 cases diagnosed yearly. However, App diagnosis as a proportion of ISU-VDL’s rapidly expanding porcine caseload has dropped dramatically from 1.2% of all respiratory diagnostic codes to just 0.47% in 2020 (internal data). Given the absence of widespread vaccination against this agent, decreased overall incidence likely reflects an effort by genetic suppliers to eliminate endemic App carriage, mostly through depopulation and repopulation of breeding herds and follow up through serology. Eradication efforts subsequently trickled down to commercial herds, resulting in decreased incidence of outbreaks. Although a recent seroprevalence survey has not been performed, there is a perception of a much lower prevalence of App in breeding herds and of the overall importance of the disease, which may have contributed to low incentivization of research into pathogen ecology and improved App surveillance tools.

The recent outbreak of App15 in finishers in IA showed that App is still a relevant disease capable of causing significant losses. Furthermore, this serotype defied clinical canon by demonstrating unexpectedly high pathogenicity, geographic and temporal persistence, and an unprecedented propensity for lateral spread. The unusual clinical course of this outbreak prompted interrogation of potential contributing management factors (biosecurity and sow farm carriage) and characterization of the causative strain of App, given its historically low-virulence serotype classification in the USA. In all, this research afforded a unique opportunity to compare ante-mortem App surveillance techniques under field conditions and to evaluate potential environmental reservoirs for the bacterium following a naturally occurring outbreak.

Longitudinal investigation of App shedding and seroconversion was performed at an affected finisher site starting 3 weeks after the initial clinical outbreak. This site experienced 29.9% mortality over the course of a single week, with a total loss of 972 pigs attributed to App15 of 4086 pigs placed.

During this investigation, App was detected by PCR in OFs up to 8 weeks after the reported outbreak. Higher detection rates were observed in the first sampling points, but over 10% detection rate was observed 8 weeks after the reported outbreak. OFs had been previously reported to have a low sensitivity for detection of App by PCR [35], only detecting 2 of 7 serotypes (serotypes 7 and 10) evaluated up to 7 days post-inoculation. Although the time of initial exposure of pigs in this study is unknown, the longer detection period observed in this study may be due to slow transmission rate of App within pens and between pens and potentially increase PCR sensitivity. Subclinically affected animals chronically harbor App in the tonsillar crypts; however, at acute stages of infection, a higher detection rate in NS was expected [22]. In this investigation, TS had a higher detection rate than NS or OFs, with positive detection in 95% of sampled animals at 8 weeks post-outbreak. Additionally, PCR of TS were overwhelmingly the most sensitive means of screening for individual carriers long-term.

Serum was collected longitudinally from the naturally infected site to monitor seroconversion. Serologic surveillance for App is complicated by serotype virulence, cross-reactivity with endemic strains, and the marked variation in sensitivity of commercially available assays [5, 23, 30]. Furthermore, investigation of natural outbreaks introduces additional variability based on the unknown rate of spread, as well as suppressive effects of treatment on seroconversion [36]. Experimental inoculation with a reference strain of App 15 has previously shown a < 70% rate of seroconversion detectable by the Swinecheck mix-App ELISA kit with evidence of waning sensitivity 6 weeks post-challenge [23]. In the longitudinally sampled site, upwards of 90% of sampled animals were seropositive 4 weeks following the reporting of the initial outbreak, although the specific infection date of individual animals is unknown. A pronounced decay of seropositivity in these same animals was noted starting 5 weeks post-outbreak; however, 30% were still positive at the final 8-week collection timepoint.

App is considered to have very poor environmental persistence, particularly under warm and dry conditions, requiring close contact with shedding animals for optimal transmission [12, 18]. This property contributes to the perception of low risk for lateral transmission between affected sites, assuming adherence to baseline biosecurity practices. Environmental detection of the novel App15 outbreak strain was investigated under field conditions to screen for pathogen-associated mechanisms of persistence and transmissibility between sites. At the affected outbreak site, App15 genetic material was primarily, if sporadically, detected in avenues of human traffic (door handles, barn entry floor) and deadstock collection sites (rendering pile). With the exception of week 8, App was not detected by PCR in any feeders or waterers. Week 8 featured an anomalous rate of environmental detection that was suspect for possible inadvertent cross-contamination during sample collection or processing. Notably, none of the PCR-positive environmental samples yielded isolates on culture. This would suggest lack of bacterial viability and/or lack of sensitivity of culture [37]; however, there was a very high degree of contamination by fecal and environmental commensals in these samples, which may have overgrown low numbers of this fastidious bacterium.

Tonsil scrapings had the highest detection rate for detection of all time points evaluated and represent the best sample type for antemortem detection of App by PCR. App was detected by PCR in oral fluids up to 8 weeks post the initial reported outbreak. Environmental detection by PCR was uncommon on surfaces investigated and bacteria viability was not demonstrated with techniques applied. Antibody-based detection of App was high at early time points, but declined over time. The results reported here generated important knowledge related to ecology and epidemiology of an unusually virulent App serotype, which can have important implications for disease diagnosis, surveillance, and biosecurity practices.

## Data Availability

The data used to support the findings of this study are available from the corresponding author upon reasonable request.
